# Spatiotemporal variation in irrigation water requirements in the China–Pakistan Economic Corridor

**DOI:** 10.1038/s41598-022-21685-4

**Published:** 2022-10-14

**Authors:** Yaqi Li, Yaning Chen, Weili Duan, Mengzhu Cao, Jingxiu Qin

**Affiliations:** 1 State Key Laboratory of Desert and Oasis Ecology, Xinjiang Institute of Ecology and Geography, Chinese Academy of Sciences, Urumqi, 830011 China; 2 University of Chinese Academy of Sciences, Beijing, 10049 China

**Keywords:** Environmental sciences, Environmental social sciences, Hydrology

## Abstract

Agricultural irrigation consumes most of the fresh water in the China–Pakistan Economic Corridor (CPEC), directly affecting water resource management and allocation. Irrigation water demand is a key component of regional water resources management. We analyzed spatiotemporal variation in irrigation water requirement, irrigation demand index (IDI), and the proposed regional optimization of irrigation water use based on the Bayesian probability network. Results showed that: (1) The IDI in the study area increased slightly (trend slope = 0.028 a^−1^) as the effective precipitation increased by 63% during this period, and total irrigation water requirement (IR) decreased from 277.61 km^3^ in 2000 to 240 km^3^ in 2015. (2) Cotton had the highest crop IDI, followed by maize and wheat. (3) According to the comprehensive scenario analysis, improving the crop planting structure (by moderately increasing the planting proportion of maize in the CPEC) is conducive to improving regional water and food security by enhancing the grain yield (+ 9%), reducing the malnourished proportion of the population (low state + 7.2%), and bolstering water-saving irrigation technologies in Pakistan as well as water conveyance systems in Pakistan. Our results form an important baseline in determining the way forward on sustainable water resource utilization management in the CPEC.

## Introduction

The China–Pakistan Economic Corridor (CPEC) is located between the Kashgar Prefecture (KP) in the Xinjiang Province of China and Pakistan and its surrounding areas. The CPEC currently represents an important part of the Belt and Road Initiative (BRI) and is essential for the promotion of regional and transregional economic and trade integration between South, Central, East, and West Asia^1^. Agriculture plays an important role and is one of the main driving forces for in national economic growth of Pakistan^2^. And Xinjiang is one of the most water stressed regions in Northwest China, which has a large area of cultivated land and is also one of the main grain producers^3^.

Water resources are an important factor limiting agricultural productivity and sustainability, which largely depend on timely and adequate water supply^4^. Most sub-regions of the CPEC are in in arid and semi-arid areas, where water resources are scarce. Pakistan is one of the poorest countries worldwide in terms of water resources. The World Bank has indicated that the renewable inland freshwater resources per capita for Pakistan are 259 m^3^, with the level of water resources available in 2018 being far less than the world (5658 m^3^) and per capita level in China (2005 m^3^) in 2018. According to the World Bank database and the Statistical yearbook of China's tertiary industry, agricultural water in Pakistan and Xinjiang accounted for more than 90% (94% in Pakistan, 93.1% in Xinjiang) of the total freshwater consumption, representing the largest proportion of water resources use in 2017. Continuous population and agricultural production growth has further exacerbated regional demand for water resources in the region^5^. In the context of scarce water resources, water supplies for agricultural irrigation are threatened, affecting crop production and human food security^6^. According to the Food and Agriculture Organization of the United Nations (FAO), the database showed that 26.3% (2019–2021) of the population in Pakistan is currently experiencing severe food insecurity. Pakistan still faces severe food security problems despite a considerable increase in the gross agricultural product (GAP)^7^. The necessity for sustainable allocation of limited water resources has become an increasingly urgent problem^8^.

Irrigation water use is essential for improving productivity of existing farmland^9^ and quantification of catchment water budgets is also fundamental to sustainably manage agricultural water resources^10^. The water consumption for crop growth and development depends on the meteorological conditions, crop types, and the crop planting area. The Penman–Monteith (PM) equation has been applied for estimating the crop reference evapotranspiration when calculating the crop water requirements. However, more meteorological data are required for broad-scale applications. Given the socio-economic and other region-specific challenges, it is difficult to obtain long-term meteorological data of high quality in Pakistan. Owing to this, we propose to use the Hargreaves–Samani (HS) formula^11^ to calculate the crop reference evapotranspiration. This formula can be efficiently applied in arid and semi-arid areas including the CPEC^12–16^. When other climate parameters are missing or have been shown to be unreliable, this formula can be used as an alternative to the PM.

A Bayesian network is the most appropriate approach for unravelling the expression and inference in the context of uncertain knowledge. It has demonstrated great potential for integrating a series of knowledge sources and has been deemed flexible for dealing with the uncertainty caused by data scarcity and/or limited knowledge of the system from a mathematical perspective^17^. Some studies have already used Bayesian networks for water resources management and in other fields by predominantly focusing on the water quality^18,19^, groundwater^20,21^, or irrigation water^22,23^. Previous studies on irrigation water requirements in the CPEC have mostly addressed to Pakistan or Xinjiang as the study area, focusing on the water demand of a single crop^24,25^. Many of these studies have lacked in-depth analysis and research on irrigation water use at the regional scale. In this study, we highlight that a Bayesian network can be applied as an effective method for addressing these problems.

This study elucidated the irrigation water requirements of the main crops of maize, wheat, and cotton in the CPEC during 2000–2015. (1) We analyzed the spatiotemporal variation of irrigation water requirements and the irrigation demand index of the main crops of maize, wheat, and cotton in irrigated areas in the CPEC. (2) We provided the scenario simulation results based on the Bayesian probability network, and (3) tailored the guidelines to support more effective agricultural irrigation management measures in the CPEC.

## Results

### Net irrigation water requirements and irrigation demand index

**Figure 1:**
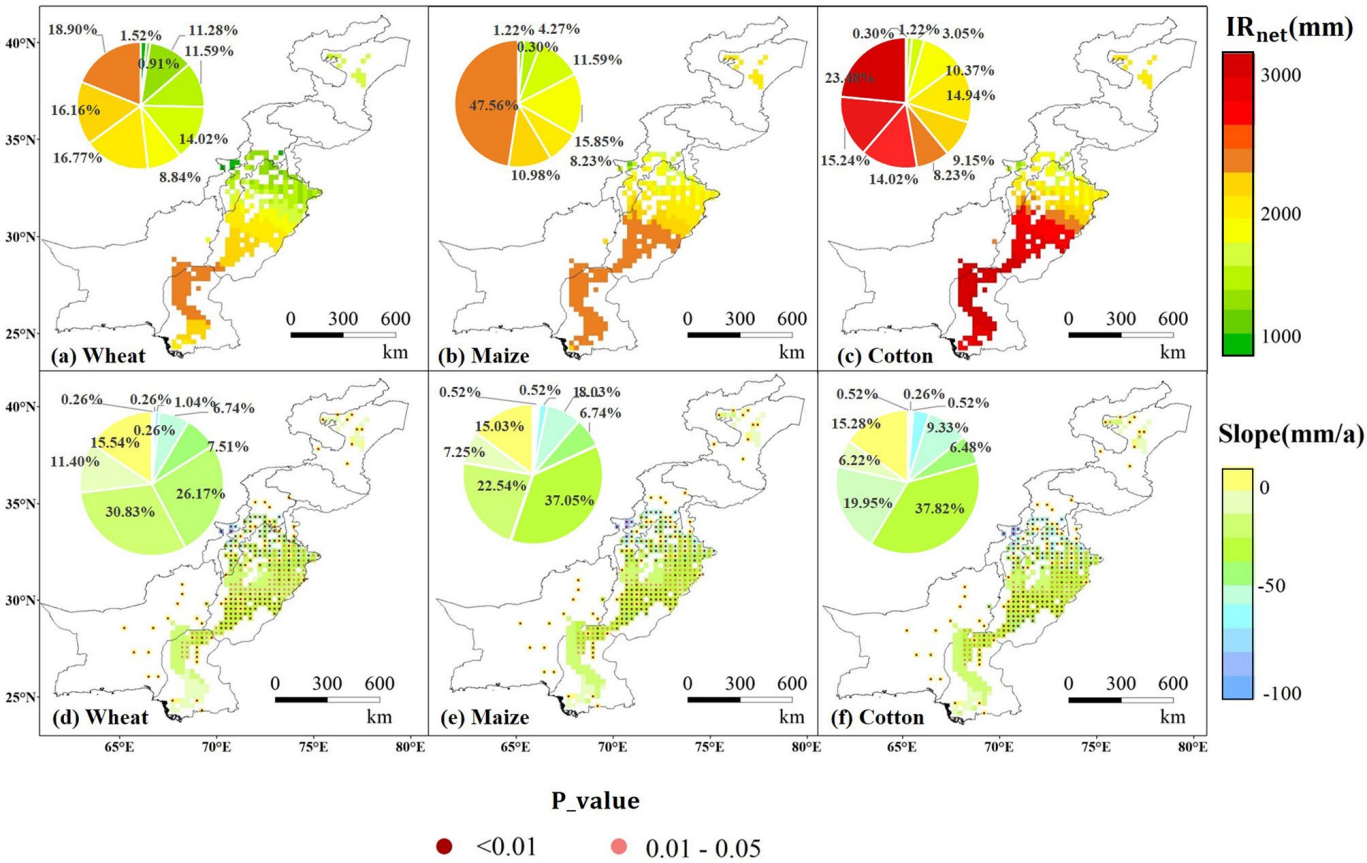
Annual average net irrigation water requirement (IR_net_) (**a**–**c**) and spatial distribution, trend and significance (**d**–**f**) for wheat, maize, and cotton in irrigated areas of the China–Pakistan Economic Corridor (CPEC) from 2000 to 2015.

**Figure 2:**
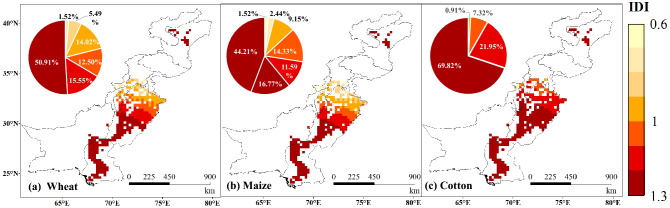
Spatial distribution of the average annual IDI of wheat (**a**), maize (**b**) and cotton (**c**) in the irrigated region of the China–Pakistan Economic Corridor (CPEC) from 2000 to 2015.

The net irrigation water requirement (IR_net_) of the three crops showed a downward trend from 2000 to 2015. Figure 1a–f shows the averaged states and its changing trend of IR_net_ for wheat, maize, and cotton in the CPEC during 2000–2015. We found that the spatial distribution of IR_net_ for the three crops was relatively consistent. The spatial distribution revealed a decreasing trend from south to north except for IR_net_ in the Kashgar Prefecture (KP), which increased slightly with a trend slope is 0–1.5 mm a^−1^. We identified that the annual IR_net_ for the three crops in descending order was: cotton, maize, and wheat. The areas with high IR_net_ for wheat were mainly clustered in the north of the Sindh province, Pakistan with the trend slope of − 10–20 mm·a^−1^. The change was found to be significant in some areas (0.01 < p < 0.05), while the lowest IR_net_ value for wheat was identified in northern Punjab, Pakistan, and the KP irrigated area in Xinjiang (< 1500 mm). The IR_net_ in some areas of the KP exhibited an increasing trend during these years, with a trend slope of 0–1.5 mm a^−1^ and a highly significant change (p < 0.01). The IR_net_ in the north of the Punjab province was estimated to be approximately 900–1000 mm with a trend slope of − 40–50 mm a^−1^. This indicates that the low value regional cluster (low IR_net_) also represented the area with the strongest decreasing trend for IR_net_. The overall distribution of IR_net_ in maize was found to be similar to that of wheat. The high-value area was in the Sindh and the south of Punjab province, where the IR_net_ was approximately 2400 mm with the trend slope of − 10–20 mm a^−1^. The low value cluster was predominantly located in the north of the Punjab province. The spatial distribution for the IR_net_ of cotton was also found to be similar to that of wheat. Areas with high IR_net_ were mainly clustered in the Sindh province and the southern Punjab province of Pakistan. This spatial pattern may be related to the intense drought in most parts of the central and southern Pakistan. In terms of the irrigation demand index (IDI), as the irrigation efficiency is considered in formula (3), its partial result is > 1. T Besides the IDI of the central and northern parts of Punjab province (< 1), the IDI of the three crops in other areas was greater than 1 (Fig. 2). The change in IDI for the three crops in these years was not significant (p > 0.5). Of them, the IDI of wheat and maize was approximately 1.0–1.2 in the middle of Punjab, 0.8–0.9 in the north, and 1.2–1.3 in other areas. The IDI for cotton was generally higher than that of wheat and maize. Besides the estimates for northern Punjab (1.1–1.2), it was found to be 1.2–1.3 in other regions, indicating a relatively high demand for irrigation. Supplementary Fig. S1 shows that the IDI for the three crops in the CPEC is relatively large, while the average annual IDI of the three crops varies and decreases, indicating that the overall irrigation demand has slightly improved for of the region with a trend slope of approximately 0.028 a^−1^. Although the overall irrigation demand of the CPEC improved slightly, the current conditions for irrigation water resources in some areas are still not sustainable. The sustainability of water resources in Punjab has decreased due to the high agricultural water consumption during the Rabbi season^26^, and there is still a severe deficit in irrigation water^27^.

### Total irrigation water requirements

#### Analysis on the change in irrigated agricultural land

During the study period (2000 to 2015), the irrigated area of CPEC showed a slightly fluctuating upward trend (Supplementary Fig. S2a). The wheat planting area was the most extensive, followed by cotton and maize. Supplementary Fig. S2c and d also illustrate that the proportion of crops being planted has varied within the CPEC. The annual average planting proportions in Xinjiang (KP and Kizilsu Kirgiz Autonomous Prefecture (KKAP)) for wheat, maize, and cotton were estimated to be 0.40, 0.31, and 0.44, respectively. The planting proportions for the three crops were relatively similar and stable during the study period. A sharp increase in the proportion of cotton planting in 2014 was recorded, which was mainly because of the government’s statistical verification of cotton planting areas, including some non-agricultural production units. In Pakistan, the planting proportion of the crops has changed to a small extent during this period. The multi-year average planting coefficients for wheat, maize, and cotton were found to be 0.46, 0.06, and 0.16, respectively.

As shown in Supplementary Fig. S2b, the change in the irrigated cultivated land area in the CPEC exhibited pronounced phased characteristics with an initial trend of continuous growth. Following this, there was a reduction in variability with an overall increase of 5398 km^2^. From 2000 to 2010, the irrigated cultivated land area in the CPEC continued to increase, until it reached 20.3 × 10^4^ km^2^ in 2010, which corresponds with the increase of approximately 13,439 km^2^. Of these, the total irrigated cultivated land area in the KKAP and KP exhibited both fluctuations along with a general increase. However, the overall fluctuation range was relatively low during this period. The change in the irrigated cultivated land area in Pakistan is in line with that of the CPEC. This suggests that the change in the actual irrigated area in Pakistan plays a key role in changing the irrigation land in the CPEC. The main driver of changes in irrigation land in Pakistan may be related to the following factor. The food crisis broke out simultaneously under the background of the global economic crisis, which has substantially affected the agricultural output value for Pakistan. Population growth and cross-border rainwater flow in Pakistan have not only offset the positive effects of grain production growth on food security but has also bolstered the import of wheat to supplement the domestic supply^28^. Pakistan has already implemented a series of measures such as increasing the cultivated land area of to increase crop yield. From 2010 to 2013, the spatial area of irrigated land under cultivation in the CPEC has decreased. Severe floods in Pakistan are potentially a key driver of this change, affected by many extreme precipitation events, which from 2010 to 2012 caused extensive crop damage and damage to approximately 2 × 10^6^ hm^2^ of farmland in 2010. This subsequently caused severe economic losses of 10 billion dollars^29,30^. Another potential influencing factor is the rapid expansion of cultivated land, which was driven by policies in the early stages of this period. The spatial area of cultivated land then rapidly expanded, vegetation cover decreased, land productivity declined, and cultivated land was abandoned by some famers. This abandonment induced a decrease in the cultivated land area due to a low level of economic efficiency^31^.

#### Analysis on changes of total irrigation water requirements of main crops

Changes in the total irrigation water requirements (IR) for the main crops during 2000–2015 are illustrated in Supplementary Fig. S3, which were obtained by multiplying the IR_net_ of the three crops and their planting areas. The IR of the three crops revealed slightly fluctuating decreasing trend with a reduction of 37.61 km^3^ and an annual average of approximately 253.99 km^3^.

We estimated the average annual IR for the three crops in the CPEC to be 153.98 km^3^ for wheat, 24.73 km^3^ for maize and 75.28 km^3^ for cotton. These results are different from the IR_net_ distribution, given that the IR is also affected by the planting area of the crops. Although the annual average estimates for IR_net_ and IDI were found to be lower than those of the other crops, wheat still has the highest IR. We found that the interannual variation trend of IR wheat was relatively consistent with the sum of the IR for the three crops. Wheat is currently the most popular food grain crop in Pakistan in its various forms is one of the main source of calorific intake for the population^32^. Wheat production accounts for the largest share of the total cultivated and produced agricultural area and is attributed to 9.1% of the agricultural added value and 1.7% of Pakistan’s gross domestic product (GDP) (Government of Pakistan Economic Survey, 2018). The change in the IR of wheat considerably affected the use of irrigation water resources. Cotton was found to comprise the lowest spatial area for planting out of the three crops, but its IR was higher than that of maize. This is predominantly because cotton is a cash crop with relatively high water consumption, and its IR_net_ is largest among the three crops. The change in the planting structure and especially the expansion of the cotton planting area, was the main driver of the significant spatiotemporal increase in the irrigation water requirements of southern Xinjiang^33^. As a typical cash crop, cotton has a substantial impact on regional water resources in the CPEC. Balancing the planting proportion of food crops and cash crops is pivotal for regional economic growth and water and food security, which emphasizes the importance for rationalizing the regional crop planting structure.

### Analysis of irrigation water use based on the Bayesian network

#### Sensitivity analysis of the key variables

The constructed Bayesian network is shown in Supplementary Fig. S4. The sensitivity analysis can be used to examine the extent to which the posterior probability distribution has changed, given the probability distribution of the other nodes in the network. This analysis demonstrated that MI and VB exhibited a relatively consistent trend and the greater the MI, the greater the VB, and the higher the sensitivity of the target variable. The impact on the target node then decreases when the intermediate variables between the node and the target node increase (Supplementary Fig. S5). Sensitivity analysis based on the evaluation method was further conducted because the applicability of the model can be verified through the evaluation versus factual evidence. In this study, five important nodes, reflecting regional irrigation water use and the security of water and food were selected as the target variables for the sensitivity analysis based on Netica software. Taking the “IR of main grain crops” and the “Effective irrigated area” as examples, the IR is the product of the crop planting area and the IR_net_ of the corresponding crops. As a result, it is most affected by the crop planting area and the IR_net_. Economic factors, grain yield, and the population, all significantly affected regional food security according to the sensitivity analysis results of “Undernourished population” which is consistent with previous findings^34^. The sensitivity analysis results are consistent with previously findings. However, since that we have not considered influencing including factors as education level, some nodes were found to be less sensitive to other nodes or could not reflect strong causal relationships.

#### Response of the target variables to scenario variables under different scenarios

The Bayesian network constructed can be used for analyzing the relative possibility of different state changes in the target variables under different management and planting scenarios. The change in each state probability for the output variable can be predicted by adjusting the state of the input variable. One of the main ideas of this study was the aim of putting forward suggestions to optimize measures for irrigation management and to adjust crop planting structures to improve the current situation of water and grain security, as well as the degree of regional cooperation. To this end, we analyzed the degree of benefit of the target variables under different scenarios to explore the changes in the target variables when each scenario variable is used as a scenario (Table 1).

**Table 1:** Responses of the target variables to the scenario variables under different scenarios.

Target node	Nodes for scenario setting													
	SA	FL	FH	WL	WH	IW	LCL	LCH	LF	LW	LM	LR	LSH	LSL
Major cash crops acreage (high)	+ 1.2					+ 0.2								
Output of major grain crops(high)	+ 0.2	+ 2.5	+ 5.6			+ 0.1			+ 5.5	+ 5.7	+ 9	+ 4.4	− 0.2	− 0.1
Major grain crops acreage (high)	+ 1.7													
Output of major cash crops(high)		+ 2.8	+ 8.7				− 1.3	+ 1.6					+ 10.7	+ 4.5
Undernourished population(low)	+ 2.1		− 0.2				− 0.2	− 0.4		+ 0.5	+ 7.2	+ 0.7		− 0.1
Undernourished population(high)													+ 0.4	+ 0.2
Arable land(high)	+ 17													
GAP (high)			+ 1.4				− 0.2	+ 0.1	+ 2.6	+ 2.5	+ 3	+ 2.2	+ 0.3	+ 0.1
Agricultural water consumption(low)		+ 0.1	+ 0.1											
Proportion of population with safe drinking water(high)		− 4.9	− 4.8	+ 0.3	− 1.4	+ 0.3			− 0.8	− 0.8	− 0.8	− 0.8		
Total IR (low)		+ 0.4	+ 0.9	− 1.4	+ 0.7				− 0.2					
Total IR_net_ of major cash crops(low)							− 5.6	− 17					− 9.6	− 4.7
Total IR_net_ of major cash crops(high)								+ 8.1					+ 4.1	
Total IR_net_ of major grain crops(high)									+ 18.2	+ 10.5	+ 9	+ 10.3		
Total IR_net_ of major grain crops(low)					+ 27	− 0.2				− 7.6	− 6.2	− 7.6		
Groundwater usage(low)				+ 8										
Groundwater usage(low)														
Applying quantity of chemical fertilizer(low)						+ 0.3								

*SA* saline-alkali land area (low), *FL* quantity of chemical fertilizer (low), *FH* quantity of chemical fertilizer (high), *WL* number of tube wells (low), *WH* number of tube wells (high), *IW* total IR (low), *LCL* cotton acreage (low), *LCH* cotton acreage (high), *LF* major grain crop acreage (high), *LW* wheat acreage (high), *LM* maize acreage (high), *LR* rice acreage (high), *LSH* sugarcane acreage (high), *LSL* sugarcane acreage (low). The “high” and “low” indicate the highest or lowest level of each node after discretization, respectively. The values in the table show the change in the percentage probability values of the specific states of the response nodes on the left after the “high” or “low” states of the upper scenario variables are determined.

As shown in Table 1, the cultivated land area can be effectively increased (+ 17%) by reducing the area of saline alkali land, compared with the actual situation. Compared with reducing the chemical fertilizer application, increasing the chemical fertilizer application within a certain range can effectively increase yield of grain crops (+ 5.6%) and cash crops (+ 8.7%) in terms of chemical fertilizer application (Table 1). However, increasing the application of chemical fertilizer would have a negative impact on safe water use (− 4.8%). Results also show that the increasing of the number of tube wells can help in improving irrigation efficiency and easing irrigation water requirements (low state + 27%). However, this also poses a threat to drinking water health (− 1.4%), which requires an urgent search for safer, more effective farming and irrigation methods to improve irrigation efficiency at the regional scale and especially for Pakistan.

In terms of the crop planting structure, three food crops were predominantly analyzed in this study, namely wheat, maize and rice. Results demonstrated that for the three main food crops, increasing the planting area for maize at the regional scale under the current scenario is more efficient for improving water and food security conditions in the CPEC. Within a certain range, increasing the planting area of maize can enhance the grain yield (+ 9%) more effectively, reduce malnourishment in population (low state + 7.2%), and increase the GAP (+ 3%). Its corresponding increase in IR (+ 9%) was less than that of the other two crops (wheat + 10.5%, rice + 10.3%). Increasing the output of the main grain crops was greater than that of the other two crops (wheat + 5.7%, rice + 4.4%). Of the cash crops, this study predominantly analyzed cotton and sugarcane. Pakistan is amongst the major sugarcane producing countries in the world. It is the world's fifth largest sugarcane producer, while Xinjiang is not a major producer of this crop. Therefore, Pakistan was predominantly analyzed in terms of the planting structure for this crop. For sugarcane and cotton, increasing a specific sugarcane planting area is more efficient for increasing cash crop yield (+ 10.7%), while the demand for IR is relatively small (+ 4.1%). Cotton is a highly important cash crop in the CPEC and the cotton area in Xinjiang is the largest cotton region in China. Cotton also considerably affects the agricultural economy of Pakistan by accounting for 8.2% of the national agricultural added value and approximately 3.2% of GDP, and approximately two-thirds of the country’s export revenue is derived from cotton and textiles^34^. However, as a crop with relatively high water consumption, increasing its planting area also induces a considerable increase (8.1%) in the IR of cash crops and affects the planting of food crops as well as regional food security. Study has demonstrated that a further expansion of the planting area has a negative impact on the development of the national economy in Xinjiang under the current high level of cotton-sown area and yield per unit area in Xinjiang^35^.

## Discussion

**Figure 3:**
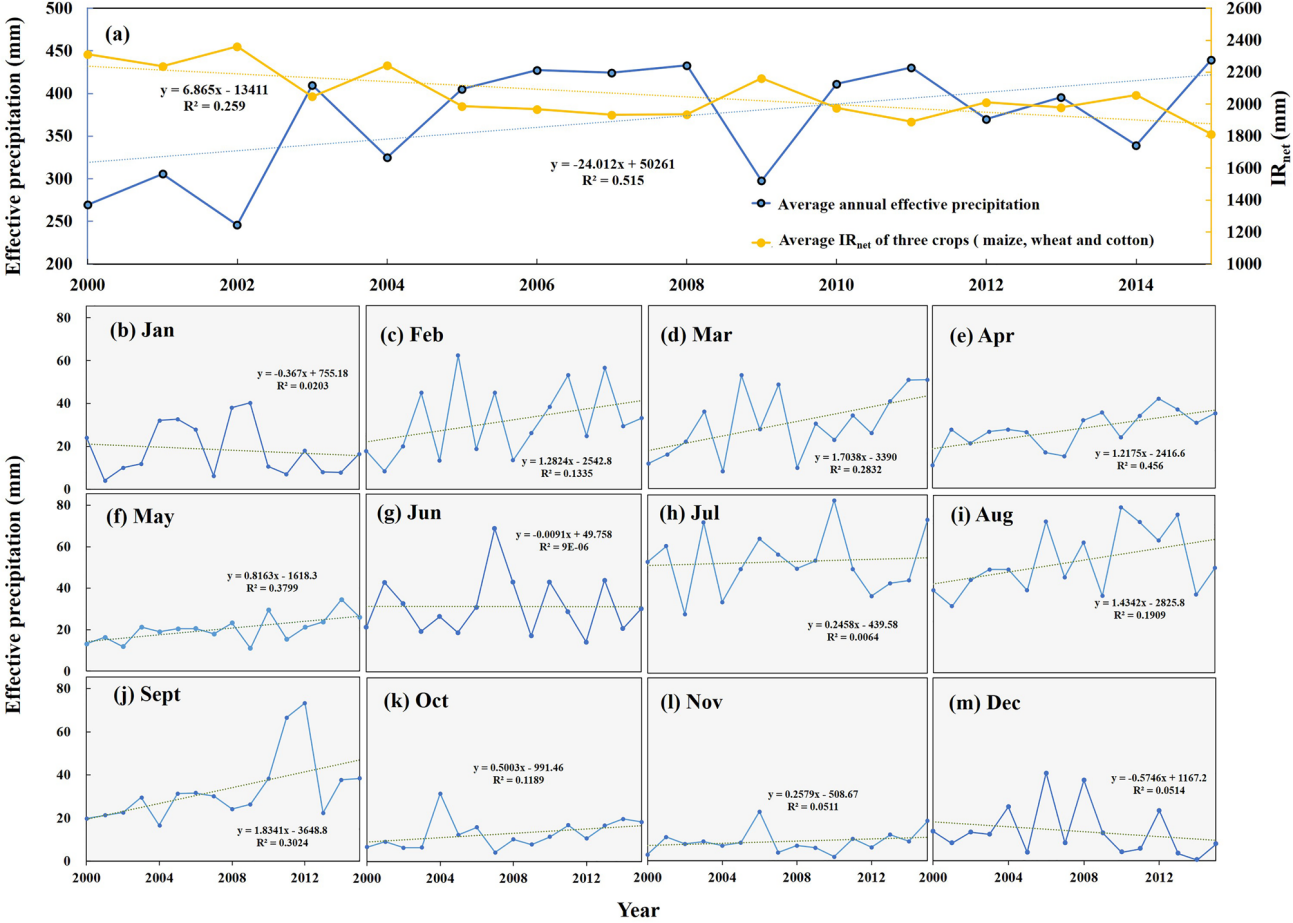
Inter-annual variation trends of the effective precipitation (P_e_) and the average net irrigation water requirement (IR_net_) for three crops (maize, wheat, and cotton) in the irrigated areas of the China–Pakistan Economic Corridor (CPEC) from 2000 to 2015.

**Figure 4:**
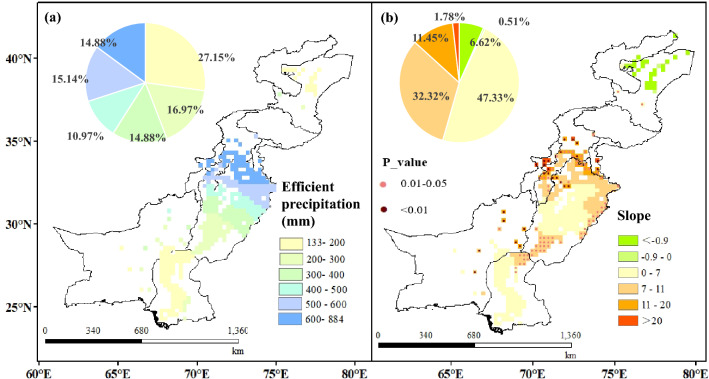
Spatial distribution (**a**), trend and significance (**b**) of the average annual effective precipitation (P_e_) in irrigated areas of the China–Pakistan Economic Corridor (CPEC) from 2000 to 2015.

Effective precipitation (P_e_) represents the replenishment of precipitation into irrigation, which is an important factor affecting the change in IR_net_. Changes in precipitation and the atmospheric CO_2_ concentration under climate change may be the main factors controlling temporal and spatial changes in IR_net_^36^. The increase of precipitation may directly lead to the decreasing trend for IR_net_^37^. The increase in P_e_ may have an impact on the decrease in irrigation water consumption in the CPEC, given that the regions along the CPEC routes are highly sensitive to climate change due to recent global warming trends^38^. As show in Fig. 3, the IR_net_ of the three crops decreased by 21%, while the annual P_e_ in the irrigated areas of the CPEC increased by 63% from 2000 to 2015. As shown in Fig. 3b–m, the annual average P_e_ in the main growth months for crops also showed a downward trend. There are spatial differences in the average annual P_e_ of irrigated areas in the CPEC (Fig. 4). The P_e_ in Pakistan showed an overall annual increasing trend, and it increased spatially from south to north, with a zonal distribution affected by altitude and terrain^39^. The northwest irrigation area of Punjab has the highest P_e_ of approximately 600 to 884 mm because it is the core region of the country for monsoon and westerlies^40^. Notably, the change in crop IR_net_ is not determined by any single climatic factor, such as the increase of temperatures and CO_2_ concentration may lead to a decrease in stomatal conductivity affecting crop transpiration to improve crop water efficiency and reduce IR_net_^41,42^. However the distribution of precipitation during the growing season has a considerable impact on the distribution of irrigation time. Optimizing the irrigation strategy according to different precipitation conditions will be an effective practice for crop production and water saving^43^, given that the increase in precipitation contributes to reducing the pressure of regional irrigation water consumption. Meanwhile, improving irrigation technologies and conveyance systems have been recognized as offering higher water saving potential^44^.

Pakistan has been forecast to have become a water-scarce country by 2025^44,45^ and water resource shortages are an acute challenge which is hampering the sustainable development of agriculture in Pakistan. According to Pakistan statistical yearbook data, Pakistan’s population reached 212.12 million in 2020, having increased by approximately 45.13% compared with 2000. Due to the population increase in population, the gap between supply and demand of water resources is increasing, exacerbating conflicts caused by water resource disputes between provinces and the international community^46^. This further underlines the importance of protecting and managing scarce resources. Future climate change scenarios have indicated that the available water and agricultural water demand in the CPEC may increase given the increase in temperature^47^. Therefore, it is pivotal to implement corresponding water-saving irrigation measures and to adjust the crop planting structure to protect oasis ecosystems and meet residents’ needs.

In Pakistan, almost all the soil is deficient in nitrogen and approximately 80% to 90% is deficient in phosphorus and 30% is deficient in potassium^48^. In the current study, the scenario analysis results indicated that an increase within a certain range can increase the crop yield, compared with reducing the amount of chemical fertilizer. However, farmers use increased quantities of fertilizers to increase yields, and consume more energy to operate modern agricultural techniques for meeting food demand^49^. Energy and fertilizer consumption intensify agricultural carbon emissions, affecting climate and natural resources, indirectly reducing the agricultural output value^50^. The use of tube wells in Pakistan has triggered a similar problem. According to the scenario analysis, it has been suggested that increasing the number of tube wells can help improving irrigation efficiency and reduce IR to some extent but it will still pose a threat to the health of drinking water. As shown in Supplementary Fig. S6, the number of tube wells in Pakistan has substantially increased since 2000 (increase = 111% in 2000–2019). Driven by the inferior quality of tube well pumping, the arsenic concentration in drinking water in the Punjab province of Pakistan exceeds the World Health Organization standard in all cases, increasing the human health risk in some parts of Pakistan^51^. Irrigation with inferior pipe well water will also lead to secondary salinization of cultivated land^52^. This further highlights the urgent necessity to implement safer, more effective farming and irrigation methods to improve irrigation efficiency in Pakistan. Being affected by climate and numerous other factors including long-term traditional farming, soil salinization in the irrigated area of the CPEC is currently severe. For Pakistan, large-scale land reclamation, based on population growth, coupled with non-scientific farming and irrigation, has led to greater salinization and a vicious circle has been formed^53^. The area of saline alkali land in the KP region is 94.2 hm^2^ accounting for 54.1% of the KP region, according to the Xinjiang Statistical Yearbook (2020), which has a considerable impact on crop planting. Mitigation of soil degradation and soil salinization are urgently required. There is also an urgent need to improve agricultural productivity, including improving the quality of crop seeds, fertilizers, pesticides, and the use of modern agricultural technologies.

According to the Xinjiang statistical yearbook, using wheat as the crop with the largest planting area in the CPEC as an example, the drip irrigation technology for wheat in Xinjiang began to be tested and popularized during 2008. By 2017, the unit yield of wheat in Xinjiang increased from 4947 kg/ha in 2008 to more than 8995 kg/ ha and, the unit yield of wheat in the KP irrigated area in 2017 also reached 6049 kg /ha. Meanwhile the unit yield of wheat in Pakistan in 2017 was 2974 kg /ha according to data from the Pakistan Yearbook. This reflects the considerable impact of the implementation of scientific and effective irrigation agricultural technology on crop yield. This also further emphasizes the importance of agricultural cooperation in the CPEC. Besides the internal coordination and optimization of Xinjiang and Pakistan respectively, it is also highly important to strengthen the cooperation between the two subregions for the future sustainable development of the region. This is expected to gradually improve regional water and grain security by strengthening exchanges in agriculture, industrial technology, and other aspects between China and Pakistan. This includes enhancing the use capacity for hydropower, to improve the efficiency of irrigation systems, and to bolster upgrading of industrial structures.

Determining the irrigation water requirements for various important grain and cash crops in the CPEC can lay a foundation for the precise estimation of irrigation water use. From the IR_net_ perspective, using HS to calculate ET_0_ as the estimated change was more sensitive to temperature compared with the PM equation^54^. Some researchers have recently developed a soil water balance simulation model to estimate the IR_net_^55^. The natural supply of agricultural water in Pakistan originates not only from rainfall, but also partly originates from ice melt water^56^. However, the calculation of IR_net_ mainly considers precipitation at present, while snow melt water was not considered. Notably, HS is relatively applicable for the CPEC and other methods can be used in future if enough meteorological data is available to quantify ET_0_. With the growing number of the relevant studies and the steadily improving data availability, the method for calculating ET_0_ will become more refined with increasing accuracy. A Bayesian probability network may highlight some hidden correlation patterns, unknown climate and/or economic development of the basin in the future, but may lack the prediction ability for determining new changes in the relationship^57^. It is challenging to evaluate factors, not included in the Bayesian networks, such as the popularity of advanced drip irrigation systems. Further work is still needed to quantify internal regimes with increased precision.

This study elucidated the spatio-temporal distribution of irrigation water requirements for maize, wheat, and cotton and analyzed the overall irrigation water use in the CPEC during 2000–2015 based on the Bayesian probability network. The main results and conclusions can be summarized as follows: (1) Overall, the area of cultivated and area with irrigation increased by 5398 km^2^ and there was a slight fluctuating decreasing trend with a reduction of 37.61 km^3^ in the total irrigation requirements for three crops in the CPEC during the study period. (2) The overall irrigation demand for the region improved slightly (the trend slope =  ~ 0.028 a^−1^), while the average annual effective precipitation increased by 71%. (3) From the perspective of crops, the IDI estimates in descending order were: cotton, maize, and wheat. These results are different from that of IR, mainly due to the influence of the crop planting area. Of the three crops, the change in IR for wheat significantly affected the use of regional irrigation water resources. The outcomes from this study could help to deepen our understanding of the spatio-temporal variation law of irrigation water demand in the CPEC area where water resources are scarce. This may also further contribute to the development of agricultural water resource management strategies.

## Methods

### Study area

**Figure 5:**
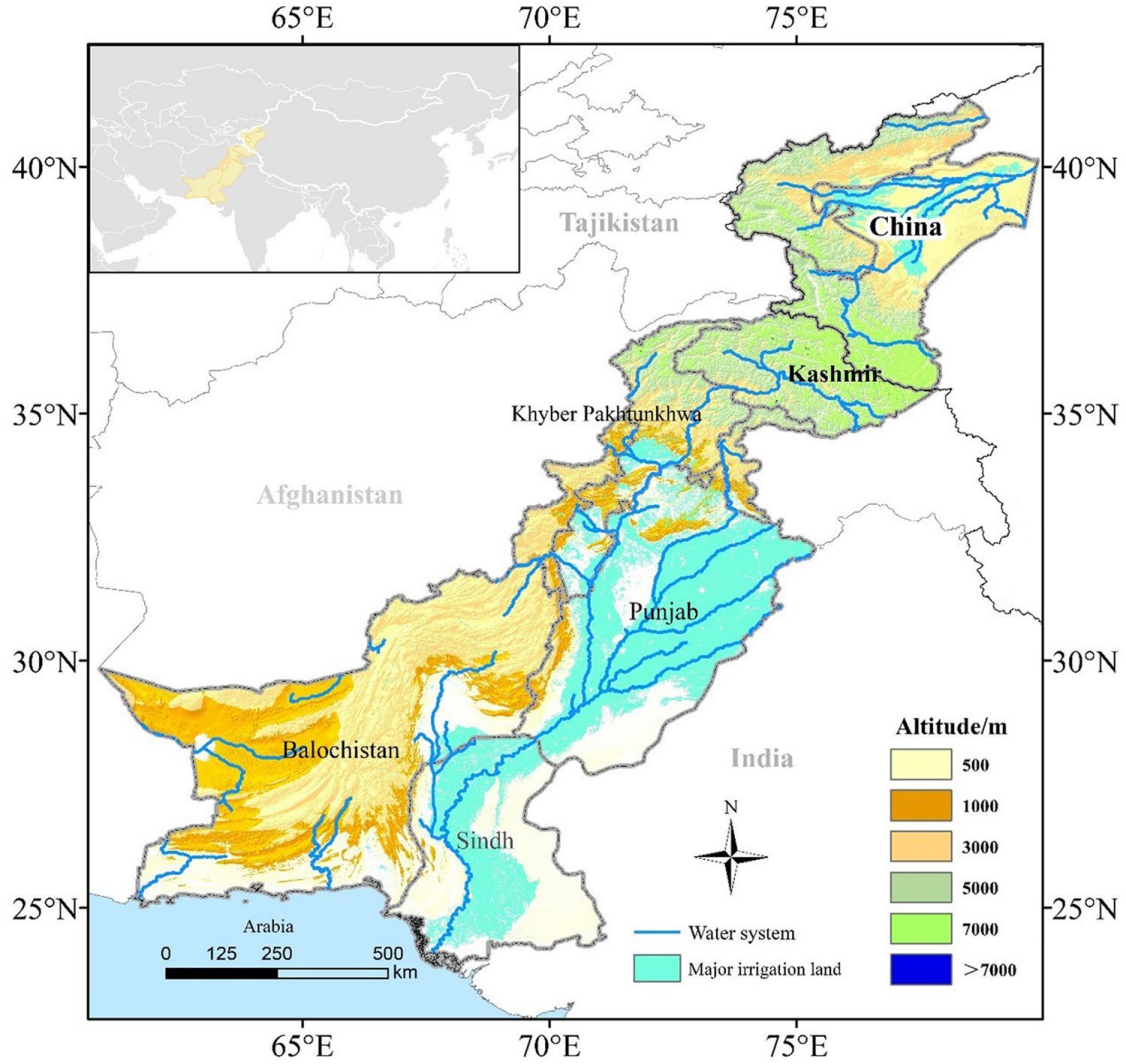
Map of the China–Pakistan Economic Corridor (CPEC) study area (Drawing approval number: GS (2020) 4619).

The CPEC is in the northwest of the South Asian subcontinent, between 24° N–40° N and 60° E–80° E. The region extends from the KP of Xinjiang province (China) in the north to the Gwadar Port in southern Pakistan. Its total length is 3000 km and its total spatial area is 927,597 km^2^. The terrain of the CPEC is high in the northeast and low in the southwest. Most of the CPEC comprises arid and semi-arid areas, while the east is considerably affected by the South Asian monsoon, with prominent dry and wet seasons. The region is highly sensitive to climate change^38^. Irrigation land in the study area is mainly distributed in the Sindh and Punjab provinces of Pakistan and KP in Xinjiang, China (Fig. 5). KP is characterized by a temperate continental climate, while Sindh and Punjab in Pakistan have tropical desert and tropical monsoon climates, respectively. The main food crops in the irrigated area of the CPEC are mainly wheat, maize and rice, while the economic crops are mainly cotton in the CPEC and sugarcane in Pakistan.

### Data sources

We used the daily resolution meteorological dataset for the CPEC from 1961 to 2015^58^, composed of observations from 65 near-ground meteorological stations in the CPEC and its surrounding areas. The final data were retrieved using the Digital Elevation Model (DEM) from the region as the covariate, with a spatial resolution of 0.25° × 0.25°. The final resolution was achieved using spatial interpolation, performed using in the ANUSPLIN (Australian National University Spline) software. In this study, the following meteorological parameters with daily resolution were used: maximum temperature, minimum temperature, and precipitation from 2000 to 2015.

Other natural and socio-economic data such as water resources and food were obtained from the Xinjiang Statistical Yearbook, Pakistan Statistical Yearbook, Pakistan Bureau of Statistics, the Economic Survey of Pakistan, the FAO Database (http://www.fao.org/faostat/zh/#data), and the World Bank Database (https://data.worldbank.org.cn/). The specific data sources are summarized in Supplementary Table S1.

### Methodology

**Figure 6:**
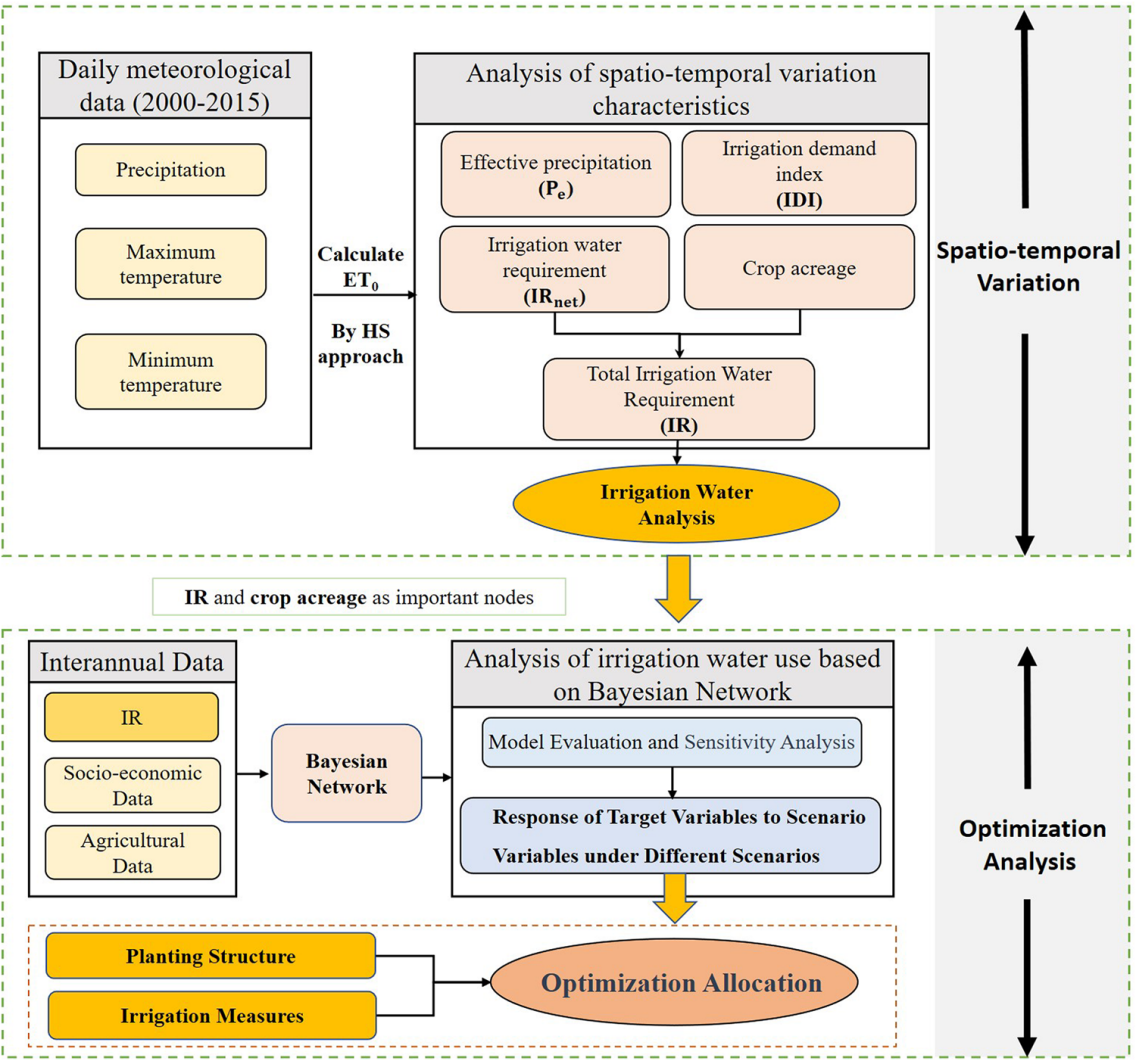
Research approach and technical route.

The main framework and the methods used in this study are shown in Fig. 6. The methods for analyzing the spatiotemporal characteristics of regional irrigation water requirements and irrigation water use are described in this section.

### Quantifying the irrigation water requirement

#### Quantifying the crop water requirement

The reference evapotranspiration (ET_0_) of crops was quantified using the Hargreaves–Samani method. ET_0_ was calculated by using atmospheric top-level radiation (R_a_) instead of solar radiation (R_s_) in this study. The calculation formula is as follows:

1 $${ET}_{0}=0.408C{R}_{a}{({T}_{max}-{T}_{min})}^{E}\cdot \left(\frac{{T}_{max}+{T}_{min}}{2}+T\right)$$

where R_a_ is the atmospheric top-level radiation [MJ/(m^2^·day)], which can be calculated from the daily ordinal number and geographical latitude, T_max_ is the daily maximum temperature (℃), T_min_ is the daily minimum temperature (℃), and C, E, and T represent the three parameters of the formula, respectively. They equal 2.3 × 10^−3^, 0.5, and 17.8, respectively.

The crop water requirement (ET_c_, mm) was calculated using the crop coefficient and ET_0_:

2 $${ET}_{c}={K}_{c}\times {ET}_{0}$$

where K_c_ is the crop coefficient. The crop coefficient refers to the estimates, as reported in previous studies^24,59–61^.

#### Calculating the net irrigation water requirement

The net irrigation water requirement (IR_net_, mm) expresses the unit water volume of crop growth dependent on irrigation in the CPEC from 2000 to 2015. According to FAO recommendations the calculation of IR_net_ is as follows:

3 $${IR}_{net}=\frac{{ET}_{c}-{P}_{e}}{{I}_{e}}$$

where I_e_ is the irrigation efficiency, which expresses the ratio of the irrigation water used by crops to the actual extracted water. According to previous studies^62,63^, the overall irrigation efficiency in Pakistan and Xinjiang Province is approximately 0.7. P_e_ is the effective precipitation, which reflects the rainfall transferred to soil moisture. In this study, we used the USDA (Unites States Department of Agriculture) method to calculate P_e_:

4 $${P}_{e}=\left\{\begin{array}{cc} P\times \frac{\left(125-0.2\times P\right)}{125} & (P <250 \, {\text{mm}})\\ 125+0.1\times P & (P\ge 250 \, {\text{mm}})\end{array}\right.$$

where P is the monthly precipitation (mm).

#### Calculating the irrigation demand index

The irrigation demand index (IDI) refers to the proportion of net irrigation water requirement to crop water requirements reflecting the dependence of crop growth on irrigation. The calculation formula is as follows:

5 $$IDI=\left\{\begin{array}{l}0 \quad { ET}_{c}\le {P}_{e}\\ \frac{{IR}_{net}}{{ET}_{c}} \quad { ET}_{c}>{P}_{e}\end{array}\right.$$

#### Calculating the total irrigation water requirement of crops

IR_net_ (mm) reflects the irrigation water required per unit area. We used the concept of total irrigation water requirement (IR) to reflect the total amount of irrigation water. The IR (km^3^) is equal to the product of the crop planting area and IR_net_, which reflects the volume of water.

### Analysis on irrigation water use based on the Bayesian probability network

#### Bayesian network

The Bayesian network is a directed acyclic graph model combining probability, statistics, and graph theory. In terms of the model evaluation and validation, sensitivity analysis has been widely deemed to be more effective in evaluating model performance^64,65^. Therefore, the sensitivity analysis was applied to evaluate the sensitivity of the Bayesian network output variables to the input variables. “Mutual information” (MI) based on entropy reduction and “Variance of Belief” (VB) based on variance reduction have been used as sensitivity analysis indicators for the Bayesian network model evaluation^66^. The calculation formulas are as follows:

6 $$MI=H\left(Q\right)-H\left(Q|F\right)={\sum }_{q}{\sum }_{f}p(q,f){\mathrm{log}}_{2}\left(\frac{p(q,f)}{P\left(q\right)P(f)}\right)$$

7 $$VB=V\left(Q\right)-V\left(Q|F\right)={\sum }_{q}P\left(q\right){\left[{X}_{q}{\sum }_{q}P\left(q\right){X}_{Q}\right]}^{2}{\sum }_{q}P\left(q|f\right){\left[{X}_{q}{\sum }_{q}P\left(q|f\right){X}_{q}\right]}^{2}$$

where V represents variance; H represents entropy; Q represents the target node; F represents other nodes; q and f represent the states of Q and F respectively; X_q_ is the real value corresponding to Q. To facilitate comparison, the mutual information and belief variance are normalized to the range of 0–100% to obtain the mutual information ratio and belief variance ratio.

#### Structure of the Bayesian network for irrigation water use in the CPEC

**Figure 7:**
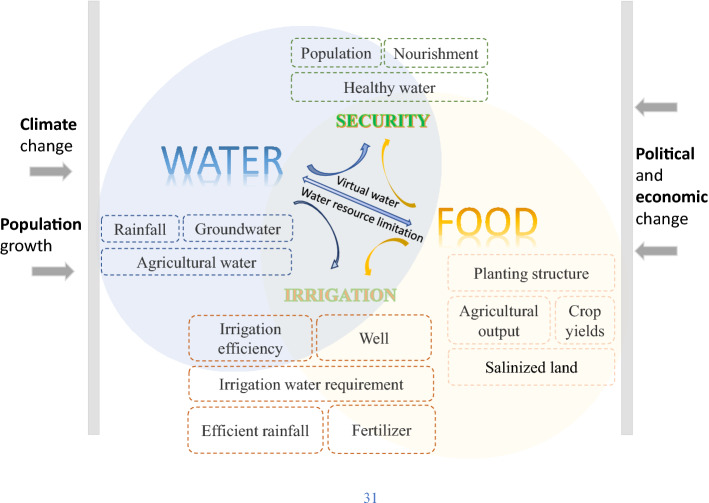
Schematic diagram of the Bayesian network structure.

A Bayesian network for irrigation water use in the CPEC was established, and its general structure is shown in Fig. 7. It is mainly composed of four modules: (1) water resource module, which reflects the changes of climate change and human activities on the available water resources, including precipitation, agricultural water and other indicators; (2) grain module, which expresses the impact of changes in water resources, planting methods and other factors on grain and its economic benefits, including indicators such as grain yield, planting structure and agricultural output value; (3) “water−food” security module, which reflects the impact of changes in water resources and food system on human health, including indicators such as the proportion of people with safe drinking water and malnourished people; and (4) irrigation module, which expresses the impact of climate change and irrigation mode on irrigation water, including effective precipitation, IR and other indicators.

In the study, we used the Netica software which has been widely used^67^ in the implementation a Bayesian networks. The expectation maximization algorithm (EM) built into the Netica software shows the probability distribution with the highest possibility through a series of step-by-step iterations based on the given data. At the same time, the data were discretized to reduce the number of calculations for the joint probability distribution in this study. The data were divided into high, medium, and low levels according to the actual conditions of each variable in the study area. This was done by using the natural breakpoint method in Netica to obtain the state classification of the node data (Supplementary Table S2).

## Supplementary Information

Supplementary Information.

## Data Availability

The datasets generated and/or analyzed during the current study are not publicly available due the data also forming part of an ongoing study but are available from the corresponding author on reasonable request.
