# Supplementary material for: Spatiotemporal variation in irrigation water requirements in the China–Pakistan Economic Corridor
Source: Sci Rep. 2022 Oct 14;12:17258. doi: 10.1038/s41598-022-21685-4 (PMC9568503; doi:10.1038/s41598-022-21685-4)
Supplement: Supplementary file 1 — Supplementary Information. [file 41598_2022_21685_MOESM1_ESM.docx]

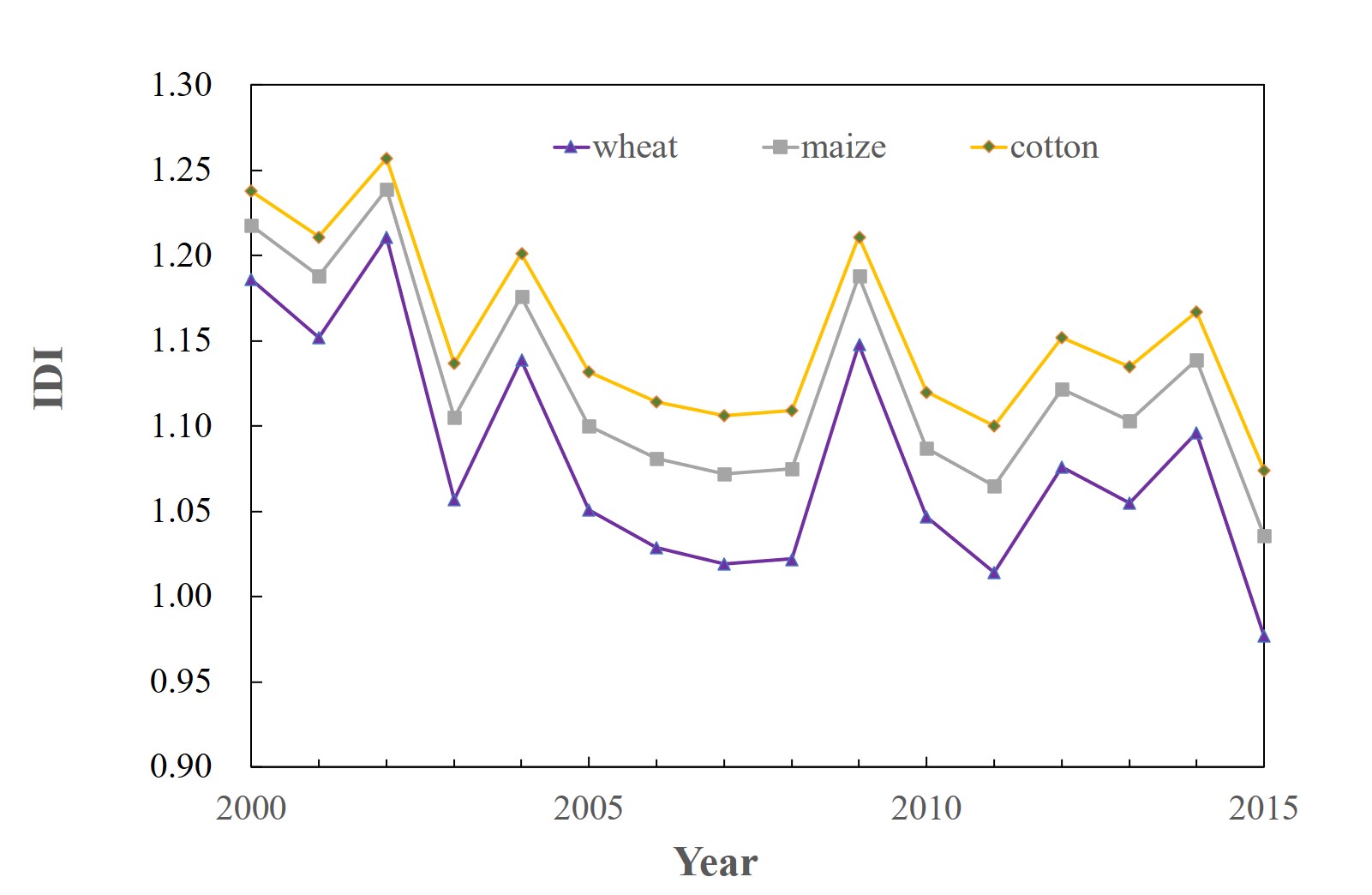
**Supplementary Figures**

**
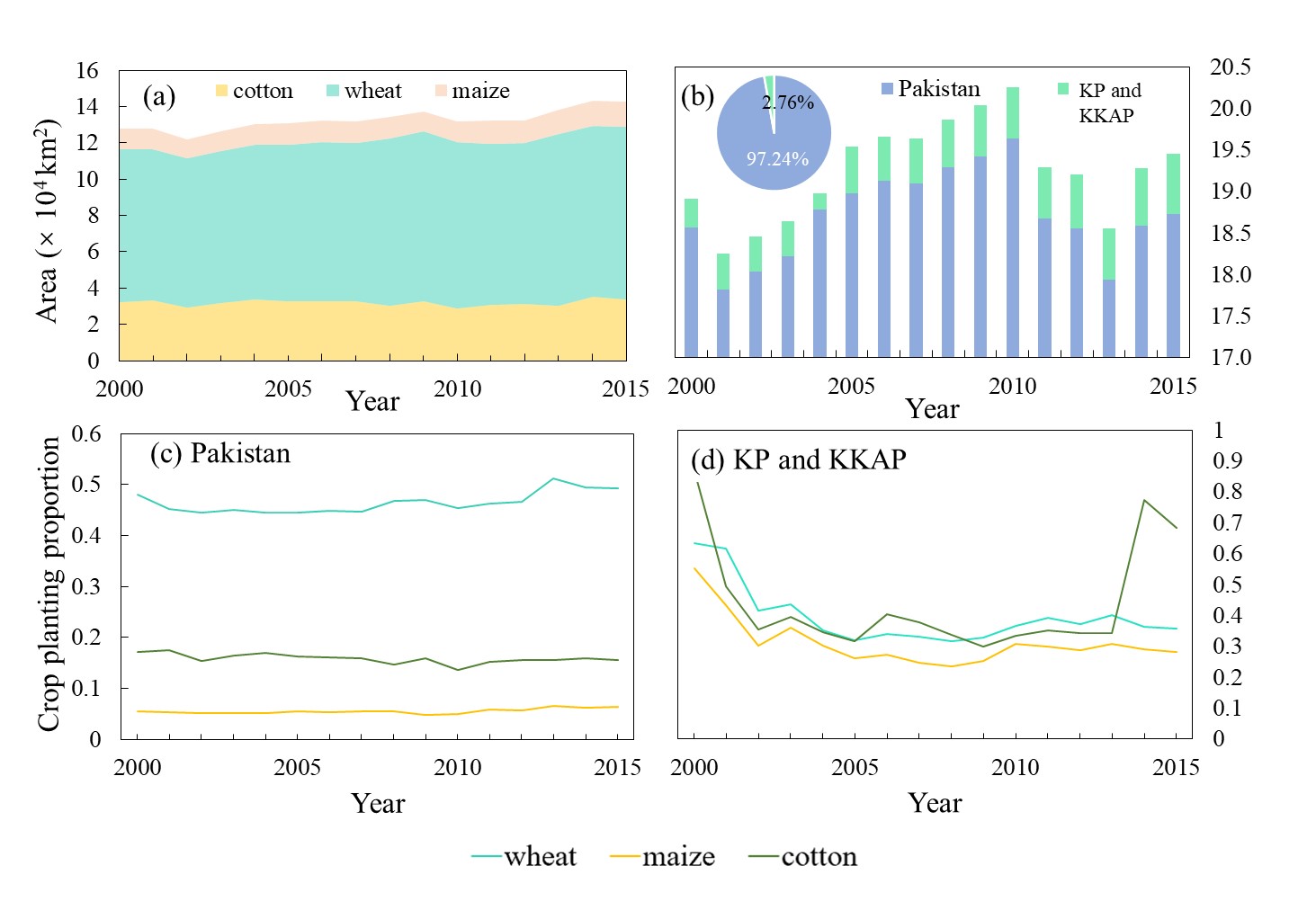
Supplementary Figure S1.** Inter-annual variation trends of IDI of wheat, maize and cotton in irrigated areas of the China–Pakistan Economic Corridor (CPEC) from 2000 to 2015.

**Supplementary Figure S2.** Changes in irrigated agricultural land of the China–Pakistan Economic Corridor (CPEC) from 2000 to 2015. (a) Crop acreage distribution of the CPEC;(b) Interannual change of actual irrigated cultivated land area of the CPEC; Proportion of three crops (wheat, maize, and cotton) planted in Pakistan (c) and Xinjiang (KP and KKAP) (d). KP: Kashgar Prefecture; KKAP: Kizilsu Kirgiz **
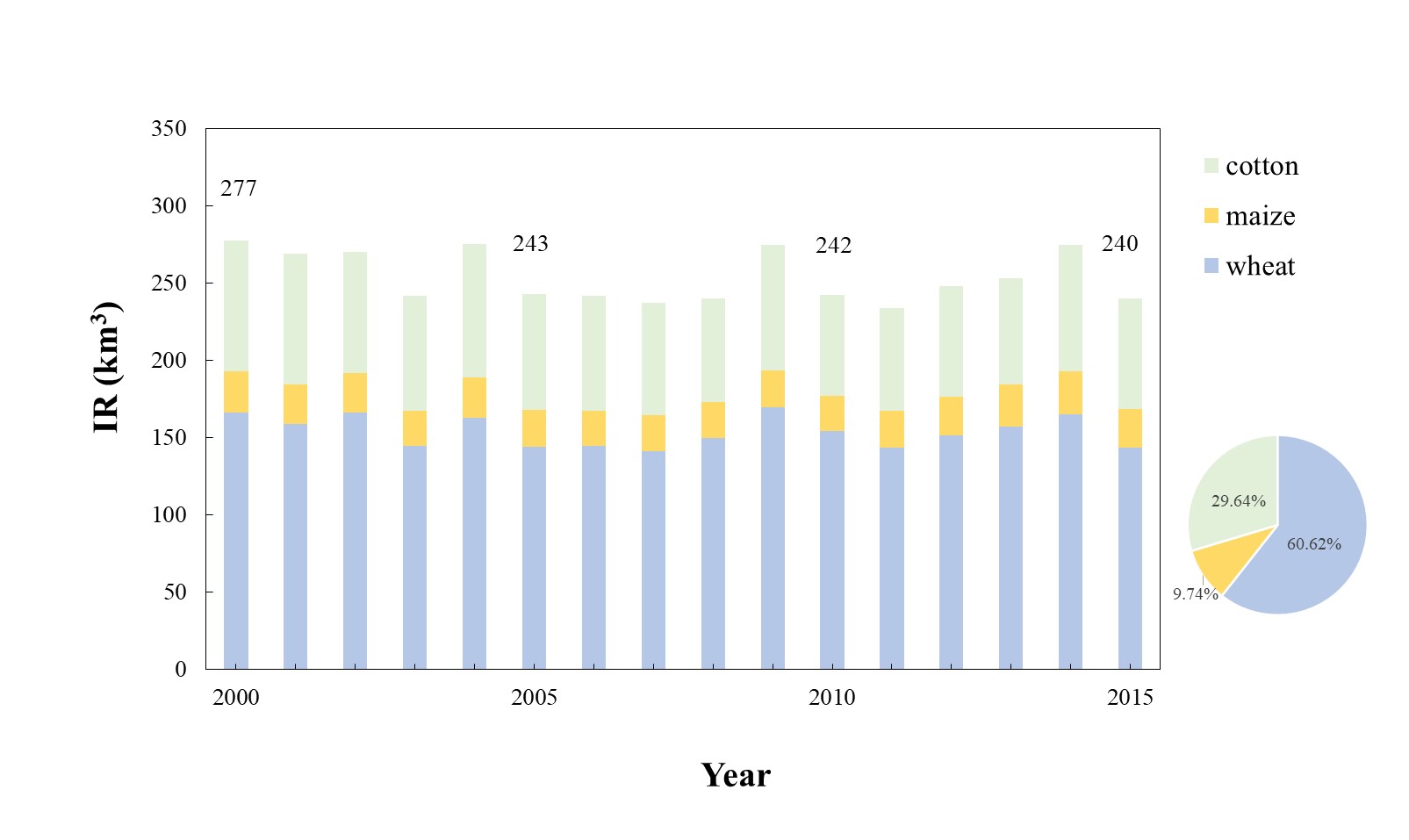
**Autonomous Prefecture.

**Supplementary Figure S3.** Inter-annual variation trend of IR of main crops in the China–Pakistan Economic Corridor (CPEC) from 2000 to 2015.

**Supplementary Figure S4.** Bayesian Network of the CPEC regional irrigation water use conditional probability calculation (2000-2015)

**Supplementary Figure S5.** Sensitivity analysis of some variables. VB: variance of belief; MI: mutual information.

**Supplementary Figure S6.** Changes in the number of tube wells and percentage of population using safe drinking water (%) in Pakistan.

**Supplementary Tables**

**Supplementary Table S1.** Data description and sources

| **Data** | **Time range** | **Description and resolution** | **Source** |
| --- | --- | --- | --- |
| Precipitation and temperature | 2000-2015 | Interpolated meteorological data set of 65 stations in CPEC,  daily scale,  0.25°×0.25°grid | A daily meteorological dataset of the China-Pakistan Economic Corridor from 1961 to 2015  (http://www.dx.doi.org/10.11922/sciencedb.j00001.00189) |
| Agriculture | 2000-2015 | Crop planting area, irrigated area and yield, etc.  Annual scale and state scale | Pakistan Statistical Yearbook, Pakistan Bureau of Statistics, Xinjiang Statistical Yearbook |
| Water use | 2000-2015 | Statistical bulletin,  annual scale and state scale | Pakistan Statistical Yearbook, Pakistan Bureau of Statistics, the Economic Survey of Pakistan, Xinjiang Statistical Yearbook and Xinjiang Water Resources Bulletin |
| Social economy | 2000-2015 | Statistical bulletin,  annual scale and state scale | Pakistan Statistical Yearbook, Xinjiang Statistical Yearbook, Pakistan Bureau of Statistics, the Economic Survey of Pakistan, World Bank database ( https://data.worldbank.org.cn/ ) |
| Water and food security | 2000-2015 | Proportion of population with safe drinking water and undernourished population,  annual scale | FAO database ( http://www.fao.org/faostat/zh/#data ) |

**Supplementary Table S2.** Detailed explanations and status discretization of some important variables

| **Variables** | **Explanation** | **Status discretization** | **Unit** |
| --- | --- | --- | --- |
| Effective precipitation | CPEC | 230-330，330-370,370-410 | mm |
|  |  |  |  |
| Groundwater use | Used in agriculture | 700-719,719-724,724-756 | 10^8^ m^3^ |
| IR of main crops | Wheat, rice, maize, sugarcane and cotton | 352-365,365-390,390-420 | km^3^ |
| IR of major grain crops | Wheat, rice, maize | 260-267,267-289,289-312 | km^3^ |
| IR of major cash crops | Sugarcane and cotton | 87-96,96-105,105-111 | km^3^ |
| Effective irrigated area | CPEC | 18200-19000,19000-19500,  19500-20300 | 1000ha |
| Output of major grain crops | Wheat, rice, maize | 23900-30100,30100-33900,3390-38100 | 1000tons |
| Output of major cash crops | Sugarcane and cotton | 45000-52000,52000-61000,  61000-70000 | 1000tons |
| Applying quantity of chemical fertilizer | Total amount of potassium, phosphorus and nitrogen fertilizer | 10000-27000,27000-32000,  32000-38000 | 1000tons |
|  |  |  |  |
| Saline-alkali land area | Pakistan | 1.69-2.1，2.1-2.4,2.4-2.7 | km^2^ |
| Proportion of population with safe drinking water | Pakistan | 35.8-36.8,36.8-37.8,37.8-38 | percent |
| Undernourished population | Pakistan | 24-28,28-31,31-33.2 | million |
| Population | CPEC | 14600-15800,15800-17700,  17700-19500 | ten thousand people |
| Number of tube well | Pakistan | 6.5-10,10-10.9,10.9-13.4 | 10^5^ |
| Agricultural water consumption | CPEC | 700-719,719-724,724-756 | 10^8^ m^3^ |
| GAP | CPEC | 9-10，10-10.8,10.8-13.5 | 10^8^CNY |
